# Pancreatic Cancer Small Extracellular Vesicles (Exosomes): A Tale of Short- and Long-Distance Communication

**DOI:** 10.3390/cancers13194844

**Published:** 2021-09-28

**Authors:** Mareike Waldenmaier, Tanja Seibold, Thomas Seufferlein, Tim Eiseler

**Affiliations:** Department for Internal Medicine, University Clinic Ulm, 89081 Ulm, Germany; mareike.waldenmaier@uniklinik-ulm.de (M.W.); tanja.seibold@uniklinik-ulm.de (T.S.); thomas.seufferlein@uniklinik-ulm.de (T.S.)

**Keywords:** pancreatic cancer, small extracellular vesicle, exosomes, sEVs, tumor growth, immune evasion, metastasis, biomarker, therapeutic sEVs

## Abstract

**Simple Summary:**

Even today, pancreatic cancer still has a dismal prognosis. It is characterized by a lack of early symptoms and thus late diagnosis as well as early metastasis. The majority of patients suffer from pancreatic ductal adenocarcinoma (PDAC). PDACs communicate extensively with cellular components of their microenvironment, but also with distant metastatic niches to facilitate tumor progression and dissemination. This crosstalk is substantially enabled by small extracellular vesicles (sEVs, exosomes) with a size of 30–150 nm that are released from the tumor cells. sEVs carry bioactive cargos that reprogram target cells to promote tumor growth, migration, metastasis, immune evasion, or chemotherapy resistance. Interestingly, sEVs also carry novel diagnostic, prognostic and potentially also predictive biomarkers. Moreover, engineered sEVs may be utilized as therapeutic agents, improving treatment options. The role of sEVs for PDAC development, progression, diagnosis, prognosis, and treatment is the focus of this review.

**Abstract:**

Even with all recent advances in cancer therapy, pancreatic cancer still has a dismal 5-year survival rate of less than 7%. The most prevalent tumor subtype is pancreatic ductal adenocarcinoma (PDAC). PDACs display an extensive crosstalk with their tumor microenvironment (TME), e.g., pancreatic stellate cells, but also immune cells to regulate tumor growth, immune evasion, and metastasis. In addition to crosstalk in the local TME, PDACs were shown to induce the formation of pre-metastatic niches in different organs. Recent advances have attributed many of these interactions to intercellular communication by small extracellular vesicles (sEVs, exosomes). These nanovesicles are derived of endo-lysosomal structures (multivesicular bodies) with a size range of 30–150 nm. sEVs carry various bioactive cargos, such as proteins, lipids, DNA, mRNA, or miRNAs and act in an autocrine or paracrine fashion to educate recipient cells. In addition to tumor formation, progression, and metastasis, sEVs were described as potent biomarker platforms for diagnosis and prognosis of PDAC. Advances in sEV engineering have further indicated that sEVs might once be used as effective drug carriers. Thus, extensive sEV-based communication and applications as platform for biomarker analysis or vehicles for treatment suggest a major impact of sEVs in future PDAC research.

## 1. Pancreatic Cancer and Intercellular Crosstalk

Pancreatic cancer is a deadly disease with a 5-year overall survival rate of less than 7% [1]. It is characterized by late diagnosis, due to the lack of early symptoms, a highly fibrotic tumor microenvironment (TME), and early metastasis [2]. The only potentially curative treatment to date is surgical resection [1]. The majority of pancreatic cancer cases (~95%) belong to the pancreatic ductal adenocarcinoma subtype (PDAC) [3]. PDAC was shown to arise from acinar-to-ductal metaplasia (ADM), induced by pancreatic injury, pancreatitis, or genotoxic events. ADMs can further evolve through acquisition of mutations into stages of pancreatic intraepithelial neoplasia (PanINs 1–3) [4]. Genetic abnormalities include early activating mutations in Kirsten rat sarcoma (KRAS) and inactivation of cyclin dependent kinase inhibitor 2A (p16/CDKN2A). Their frequencies increase with dysplasia. Mutations in tumor protein 53 (TP53) and SMAD family member 4 (SMAD4) inactivation are late events observed in PanIN3 lesions, which in the end develop into full-blown PDAC with a highly fibrotic and complex TME [4,5,6]. The TME comprises cancer-associated fibroblasts (CAFs), pancreatic stellate cells (PSCs), and immune-inhibitory cells, such as immunosuppressive tumor-associated M2-polarized macrophages (M2-TAMs), regulatory T-cells (T_regs_), or myeloid-derived suppressor cells (MDSCs) [7,8]. Within the TME, tumor cells were shown to communicate with the surrounding stromal cell compartment through the release of secreted factors, but also via extracellular vesicles [9]. In the recent years, in particular small extracellular vesicles (sEVs, exosomes) were described as major mediators of intercellular communication during cancer initiation and progression [10]. In fact, tumor cells do not only communicate with cells in the primary TME, but there is also long-distance communication via circulating sEVs, e.g., during the establishment of pre-metastatic niches (PMNs) [11]. Thus, research into sEV biogenesis and function has evolved as a promising new research field that helps to define novel mechanisms of tumor evolution.

## 2. Overview: Small Extracellular Vesicles and Its Predominant Subgroup Exosomes

The term sEVs specifies a particular subgroup of extracellular vesicles with a diameter of 30–150 nm, which is predominantly made up by the exosome subgroup, although a subpopulation of microvesicles has also been described in the respective size range [12]. This review mainly focuses on the role of exosomes in PDAC. For the sake of easy communication with the reader, we have however attributed biological effects to the broader specification “sEVs”, which is often used instead of the term “exosomes” in the literature [13]. sEVs are formed as intraluminal vesicles (ILVs) in endosomal-derived multivesicular bodies (MVBs). Upon transport and fusion of MVBs with the plasma membrane, the intraluminal sEVs are released into the extracellular space [14]. Initially, sEVs were thought to be vehicles for cellular waste removal. However, further research revealed that a major function of sEVs is intercellular communication [15]. sEVs are present in almost all body fluids, i.e., blood, saliva, urine, liquor and many more. In electron microscopy, sEVs mainly present as spherical cup-shaped nanoparticles, engulfed by a phospholipid bilayer [16]. They typically contain numerous bioactive molecules, including proteins, various nucleic acids and lipids as well as inorganic substances, which are locally and systemically transferred to recipient cells [17]. sEVs are generated by different cell types under physiological and pathophysiological conditions that critically shape the respective cargo profile [18]. sEVs share similar structural proteins, such as Rab-GTPases, class 1 and 2 major histocompatibility complexes (MHC I/II), annexins, ALG-2 interacting protein X (ALIX), tumor susceptibility gene 101 protein (TSG101), flotillin (FLOT1), integrins, and in particular tetraspanins (Tspans), which are major surface markers enriched in sEVs [19,20]. Tspans belong to a 4-transmembrane protein family, comprising CD9, CD63, CD81, CD82, CD53, and CD37, which are up to a 100-fold more enriched in sEVs compared to their parental cells [21,22]. Tspans can form homo-and heterodimers, as well as complex secondary and tertiary interactions known as the tetraspanin web. They couple to lipids (e.g., cholesterol) and form tetraspanin-enriched microdomains (TEMs), which help to recycle Tspan binding partners, such as specific integrins or proteases from the cell surface into MVBs and eventually sEVs [23,24]. In addition to TEMs, sEVs also contain caveolae lipid raft microdomains. Both structures help to transduce important signals, such as apoptosis and cell cycle arrest, via lipid molecules or resident proteins [25,26,27]. Several lipids, such as cholesterol, sphingomyelin, gangliosides, ceramide, phosphatidylserine, and phosphatidylethanolamine, make up the composition of sEV membranes, which can also have important signaling properties dependent on the respective cellular context [28,29]. sEV cargo also contains a broad spectrum of nucleic acids, including messenger (m)RNA and noncoding (nc)RNAs, such as micro (mi)RNA, ribosomal (r)RNA, transfer (t)RNA, long non-coding (lnc)RNA, long intervening non-coding (linc)RNA, small nuclear (sn)RNA, small nucleolar (sno)RNA, circular (circ)RNA as well as cell-free cellular (cf)DNA or mitochondrial (mt)DNA [30]. In cancer, sEVs were shown to be highly enriched in miRNAs [31,32]. The sEV proteome comprises signal intermediates, heat shock proteins, such as HSP70/90, as well as epithelial cell adhesion molecules (EpCAM), cell membrane receptors, e.g., EGFR and other human epidermal receptor (HER) family members, immunomodulatory proteins, cytokines, cytoskeletal molecules, and cytosolic components [20,33]. 

### 2.1. sEV-Biogenesis

sEV-biogenesis starts at the endosomal compartment by maturing early endosomes into late endosomes or MVBs, where membranes invaginate to generate ILVs. The formation of ILVs is facilitated by two major pathways: The endosomal sorting complex required for transport (ESCRT)-dependent and ESCRT-independent pathway. The ESCRT machinery is a large multi-protein complex consisting of four subcomplexes: ESCRT-0, -I, -II, -III as well as the associated AAA-ATPase Vps4, which initiate biogenesis in a coordinated fashion [34]. ESCRT-0 is thought to initiate the pathway by binding to phosphatidylinositol-3-phosphate (PI3P) and clustering tagged, ubiquitinated membrane EV cargo proteins. Then ESCRT-I is recruited by ESCRT-0, which also binds to the ubiquitinated cargo, followed by ESCRT-II [35,36]. The ESCRT-II subunit Vps25 subsequently serves as a nucleation hub for the stepwise assembly of a filamentous ESCRT-III complex, which in turn facilitates cargo sequestration and inward budding of the ILV. Afterwards, ESCRT-III also terminates the assembly of the filaments on the endosome surface [37,38]. Then, AAA-ATPase Vps4 is recruited and catalyzes the disassembly of the ESCRT-III filaments in an ATP-driven reaction to terminate MVB-biogenesis, followed by the release of the cargo-laden ILV [39]. MVB-biogenesis can also progress without ESCRTs. Tspans and ceramide are involved in ESCRT-independent sEV-biogenesis and -release. Inhibition of neutral sphingomyelinase 2 (nSMase2), an enzyme that generates ceramide from sphingomyelin, by the small molecule GW4869 [18,40,41,42] has been shown to reduce sEV-release [18,40]. ESCRT-dependent and ESCRT-independent mechanisms might also not be entirely separated. Both pathways could work synergistically, and different subpopulations of sEVs may use different machineries. Additionally, cell type or cellular state are important factors determining the type of vesicle biogenesis [43]. Once ILV-biogenesis is complete, MVBs have to be transported along microtubules to the plasma membrane (PM), where ILVs are released upon fusion [44]. This process is controlled by soluble N-ethylmaleimide-sensitive factor attachment protein receptors (SNAREs) and small Rab-GTPases, such as Rab27a/b or Rab11, which regulate different aspects of ESCRT-dependent and -independent sEV release [21]. Moreover, efficient fusion and sEV release from cells requires the presence of branched actin filaments at the PM. Branched actin is stabilized or debranched by the antagonistic action of the actin-regulatory proteins Cortactin and Coronin-1, respectively [45]. Cortactin is also involved in the Arp2/3-complex-dependent synergistic nucleation of branched actin filaments at the PM together with the nucleation promoting factor WAVE2 [46,47]. In PDAC cells, knockdown of WAVE2 and Cortactin critically impaired sEV release. An additional layer of control is added by a posttranslational modification of Cortactin that only contributes to actin-mediated sEV-release when a regulatory phosphorylation by Protein Kinase D (PKD) is abrogated [47,48]. Under more specialized conditions, such as when cancer cells form invadopodia, N-WASP, a different nucleation promoting factor, is required for synergistic nucleation and sEV secretion [45]. Thus, sEV-biogenesis is a highly regulated and coordinated process and secretion as well as final release of different sEV subpopulations can be dependent on different biogenesis pathways. 

### 2.2. sEV-Uptake and Reprogramming of Recipient Cells

Upon delivery of sEVs to recipient cells, e.g., by blood flow, sEVs interact with the respective cells in different ways. They can directly bind via surface ligands to membrane-integrated receptors and thus activate specific signaling pathways. Alternatively, sEVs are internalized to release their transported cargos, which is facilitated by various mechanisms, including direct membrane fusion, clathrin- or lipid raft (Caveolae/caveolin-1)-mediated endocytosis, micropinocytosis, or phagocytosis. Upon entering a cell, e.g., by endocytosis, late endosomes containing sEVs either fuse with lysosomes to recycle sEVs and their cargos, or release their content into the cytoplasm to trigger signaling pathways or transcriptional changes [21]. There is ample evidence that sEVs have vital functions in carcinogenesis and evolution of PDAC as well as in the pathogenesis of precancerous conditions of the pancreas, including pancreatitis or pancreatic fibrosis [49,50]. Indeed, sEVs were shown to promote the transformation of precancerous lesions, such as PanINs to PDAC. They extensively contribute to intercellular communication between tumor cells and associated cells in the primary TME, facilitate cell migration, epithelial-to-mesenchymal transition (EMT) as well as apoptosis and chemoresistance. In later stages they also impact on PDAC metastasis by inducing the establishment of organ-specific PMNs [50]. Here we focus on the functions and underlying molecular mechanisms that are involved in sEV-mediated PDAC carcinogenesis, tumor progression, and metastasis.

## 3. Modulators of sEV-Biogenesis

PDACs are often associated with elevated sEV secretion, as plasma samples acquired from PDAC patients show enhanced concentrations of circulating sEVs, which are further increased with metastatic burden [51]. Different cellular and molecular stress conditions, such as hypoxia and low pH can foster formation of sEVs and cause quantitative, but also qualitative changes in the sEV cargo content. sEVs were therefore described as promising analytes to evaluate the presence of diagnostic and prognostic markers [52,53]. Indeed, hypoxia is also an important characteristic of the PDAC TME and was shown to trigger the release of sEVs with smaller size, which help the tumor to adapt to challenging conditions and enable survival of tumor cells [54]. Moreover, oncogenes facilitate sEV-biogenesis in different cancer entities, altering sEV concentration, size, and cargo [55,56,57,58,59,60]. They also maintain biomass homeostasis and foster accelerated cell division as well as tumor growth by enhanced sEV-biogenesis. Moreover, oncogenes, such as Harvey rat sarcoma (HRAS), aurora kinase B (AURKB), and MYC were shown to promote aberrant sEV secretion by triggering hyperactivation of ESCRT-pathways, the ceramide metabolism, or by reducing lysosome-associated gene expression, which also shifts the protein and miRNA content of sEVs [55]. In cancer, sEVs are highly enriched in miRNAs [31,32]. There is evidence, that miRNAs influence oncogenic processes by either suppressing or promoting the expression of oncogenes (tumor suppressor miRs or oncomiRs) [61]. The KRAS oncogene drives PDAC carcinogenesis and not only promotes sEV-release, but also alters their cargo composition compared to wild-type KRAS tumors. The mutant KRAS-derived sEVs are characterized by tumor-promoting proteins, including mutant KRAS and p53 as well as an altered miRNA content, enabling oncogenic transfer and metabolic reprogramming in recipient cells [58,59,60]. Thus, oncogenes modulate sEVs and are horizontally transferred via sEVs to surrounding cells [51,55,62]. So far in PDAC, no oncogenes were directly described to boost sEV-biogenesis. Since mutations in KRAS are found in >90% of PDACs and mutated KRAS facilitates changes in sEV cargo content in colorectal cancer, similar functions are however very likely [55,59,63].

## 4. sEVs in Pancreatic Cancer Initiation

Pancreatitis is considered a risk factor for the development of PDAC. In addition, tobacco smoking, diabetes, obesity, physical inactivity, infections, genetic alterations, and alcohol consumption can contribute to PDAC carcinogenesis [64]. Pancreatitis is classified into acute (AP) or chronic (CP), as well as autoimmune (AIP) pancreatitis, whereby the latter is a type of CP with very distinct histological and clinical features [65]. In particular CP carries an increased risk for the development of PDAC [66,67]. Pathology of CP includes exocrine and endocrine pancreatic insufficiency, inflammation as well as high levels of pancreatic fibrosis [68]. There has been increasing evidence that sEVs are also involved in inflammatory signaling during pancreatitis or carcinogenesis of PDAC [69]. During AP, the concentration of circulating sEVs is significantly increased. The respective sEVs originate from liver and immune cells and mediate molecular changes associated with irreversible interstitial fibrosis as well as parenchymal pancreatic calcification. Moreover, the respective sEVs pass through the endothelial barrier in lungs and induce M1-polarization of macrophages promoting acute lung injury (ALI) [70]. Interestingly, circulating AP-sEVs also contain proinflammatory miRNAs such as miR-21/122/155 [71]. CP also causes substantial pancreatic tissue destruction as well as exocrine and endocrine insufficiency. It results in the activation of PSCs, inducing their proliferative capacity [72]. PSCs in turn communicate with pre-cancerous PanINs to promote their progression [73,74]. This is mediated by connective tissue growth factor 2 (CCN2/CTGF2). Clinical studies have also demonstrated that CCN2 is highly expressed in PSCs from (alcoholic) CP patients [75]. CCN2 expression is controlled by miR-21 and both CCN2 as well as miR-21 were detected in PSC-derived sEVs. Using an alcoholic pancreatitis mouse model it has been shown that miR-21- and CCN2-positive sEVs educate other PSCs in a paracrine fashion to potentiate proliferation and collagen deposition [76].

## 5. sEV-Mediated Crosstalk of PDAC and Associated Cells in the TME

Upon progression through PanIN stages, carcinogenesis is concluded with the development of full PDAC tumors, which shape their surrounding TME by interacting with stromal extracellular matrix (ECM) components and cells, such as stromal fibroblasts or cells of the innate and adaptive immune system [77]. A major hallmark of PDAC is the desmoplastic tumor stroma, which emerges from abundant ECM deposition that can constitute up to 90% of the tumor mass [2,78]. The TME comprises various cell types, e.g., fibroblasts, mesenchymal cells (MSCs), and immune cells [79]. The non-cellular components include ECM proteins, such as collagen, fibronectin, hyaluronic acid, laminin as well as metabolites, cytokines, and growth factors [80,81]. Autocrine and paracrine interactions between the different cell types and the tumor cells extensively contribute to PDAC tumorigenesis, angiogenesis, metabolic reprogramming, impaired antitumor immune responses, drug resistance, and metastasis [82,83,84]. Over the last years, sEVs were shown to critically contribute to PDAC carcinogenesis and progression, enabling intercellular crosstalk between the tumor and surrounding cells in the TME, e.g., by triggering the transformation of non-malignant to malignant cells [82,85]. A comprehensive overview of the respective sEV cargos involved in PDAC crosstalk with the TME is available in Table 1.

### 5.1. PDAC-sEVs and CAFs

The TME consists of matrix-associated cell types, which utilize sEVs to interact with PDAC tumor cells and vice versa tumor cells were shown to reprogram associated stromal cells, e.g., fibroblasts [9,50]. During early tumor initiation, tumor-derived sEVs reprogram PSCs to direct their differentiation into CAFs via TGFβ/Smad signaling [85,86]. Moreover, CAFs can also be generated from normal fibroblasts [87] and represent one of the most prominent and heterogenous components of the TME. They mediate pro- and antitumorigenic functions but are mainly responsible for the extensive desmoplasia associated with PDAC. Aberrant ECM deposition and remodeling associated with massive desmoplasia further causes hypoxia and blood vessel depletion, triggering alterations in blood supply and thus metabolic adaptation of tumors, which eventually foster PDAC aggressiveness [80,85,88,89]. Moreover, oxygen deprivation was demonstrated to promote sEV-biogenesis, as PDAC cells release increased amounts of sEVs with smaller size to ensure survival under such conditions [54]. In addition, hypoxia is associated with changes in sEV cargo content, e.g., by facilitating the secretion of miR-301a-3p-loaded sEVs from PDAC cells. Paracrine transfer of these sEVs to other PDAC cells enhanced tumor cell invasiveness and uptake by macrophages mediated conversion to immunosuppressive M2-subtypes [90]. Additionally, PDAC cells were reported to benefit from CAF-derived sEVs under nutrient-stress conditions. Here, CAF-sEVs enhanced the Warburg effect in PDAC cells by reprogramming the energy metabolism through direct delivery of de novo metabolites to support the entire carbon metabolism and PDAC survival [91]. Metabolite transfer by sEVs was also shown using ^13^C metabolic flux analysis to track dynamic changes in cargo release from CAFs and internalization of sEVs by cancer cells over time [92]. Other consequences of hypoxia are reduced sensitivity towards radio- and chemotherapy as well as immunosuppression [89]. 

### 5.2. sEV-Based Crosstalk of PDAC and PSCs

PSCs mediate vital functions during pancreatic fibrosis [93]. Their interaction with tumor cells and stromal cell components enhances cell growth and distant metastasis [94]. PSCs usually exist in a quiescent state and maintain normal stromal composition (ECM turnover). Their activation in the TME is achieved by stimuli, such as environmental stresses or secretory proteins, e.g., growth factors and cytokines, which induce mitogen-activated (ERK) and Jun kinase (JNK) signaling. Upon activation, PSCs are transformed into different CAF subtypes that are major regulators of tumor-stromal crosstalk [95,96]. Once activated, PSCs further secrete factors, which promote activation of quiescent PSCs in a feed-forward loop, and this is facilitated in part through paracrine transfer of sEVs containing CD9, CCN2 and miR-21 cargo, driving fibrosis [76,97,98]. In addition, PDAC cells induce proliferation and migration of PSCs by transfer of sEVs containing miR-1246 and miR-1290 to upregulate α-smooth muscle actin (α-SMA/ACTA2) as well as procollagen type I C-peptide (PIP) via ERK and Akt signaling [99]. On the other hand, PSC-derived sEVs were reported to influence PDAC cells by stimulating chemokine expression (C-C chemokine ligand 20, CCL20; C-X-C chemokine ligand 1 and 2, CXCL1/2), fostering tumor cell proliferation and migration [98,100]. PDAC proliferation was also promoted through the sEV-based transfer of miR-5703, which targets CKLF-like MARVEL transmembrane domain containing 4 (CMTM4), resulting in the activation of the PI3K/Akt pathway by p21-activated kinase (PAK4) [101]. Thus, PSCs and PDAC cells are engaged in an extensive crosstalk utilizing sEVs to enable tumor progression.

### 5.3. sEVs in Angiogenesis

Abundant ECM deposition and extensive fibrosis in the TME can implement a mechanical barrier. This prevents tumor cells from acquiring sufficient oxygen and nutrients, thereby limiting tumor growth. The hypoxic conditions also trigger the release of pro-angiogenic molecules, such as vascular endothelial growth factor (VEGF) from tumor cells to facilitate angiogenesis. Angiogenesis is a multistep process to generate new blood vessels from preexisting ones [102], thus enabling survival, growth, and metastatic spread of tumors. Pancreatic cancer is characterized by high microvascular density and concomitantly impaired microvessel integrity. These blood vessels are poorly perfused and display a heterogenous distribution in different subtypes [103,104]. The combination of both parameters has been associated with early recurrence, metastasis, and short survival after tumor resection [105]. PDAC-derived sEVs contain several cargos that support angiogenesis by activating surrounding stromal cells to induce ECM remodeling as well as neovascularization [106]. In a rat PDAC model, incubation of endothelial cells (ECs) with PDAC-derived sEVs, harboring Tspan8, CD106, or CD49d (Integrin α4) triggered the expression of pro-angiogenic factors, including von Willebrand factor (VWF), TSPAN8, CXCL5, migration inhibitory factor (MIF), C-C chemokine receptor type 1 (CCR1), VEGF, and VEGFR2. This reprogramming induced EC proliferation, migration, sprouting, progenitor maturation and thus neovascularization, independently of VEGF-driven angiogenesis [107]. In addition, PDAC cells were shown to release VEGF-C containing sEVs upon downregulation of the dual-specificity phosphatase-2 (DUSP-2), promoting lymphovascular invasion [108]. Of note, VEGF-C was also associated with vasculogenic mimicry by tumor cells, which is a formation of blood vessel-like structures independent of angiogenesis by endothelial cells. A similar phenotype was reported for Ephrin Type-A Receptor 2 (EphA2) signaling, a sEV-resident biomarker in PDAC [109,110]. More sEV cargos involved in the regulation of angiogenesis are summarized in Table 1.

### 5.4. Immune Cells in the TME

A major feature of the PDAC TME is the immunosuppressive cellular environment that is able to inhibit innate and adaptive immune responses [111]. Antitumor immunity is triggered by the release of tumor-associated antigens (TAAs) and activation of immune effector cells, such as natural killer (NKs) and CD8+ T_effectors_ [112]. The PDAC TME harbors a large amount of immunosuppressive cell types, such as T_regs_, M2-TAMs, and immature myeloid-derived suppressor cells (iMDSCs), which inhibit proper CD8+ T-cell responses, functional antigen presentation/lymphocyte activation by dendritic cells (DCs), or the anti-tumor response by M1 macrophages (M1-TAMs) [113]. A vital part of the immunosuppressive signaling in the TME is mediated by sEVs, e.g., by facilitating the transformation of immune cells into immunosuppressive and pro-tumorigenic phenotypes [111]. This helps tumors to bypass immune surveillance by facilitating functional losses in lymphocytes or inhibiting lymphocyte activation and survival [114]. sEVs are involved in the suppression of both innate and adaptive immune responses [115].

#### 5.4.1. Innate Immunosuppression and Tumor Associated Macrophages

TAMs are critical components of the TME [116]. Macrophages are involved in numerous biological processes including tissue homeostasis, defense against pathogens and wound healing [117,118]. They originate from circulating monocytes and are transformed at sites of inflammation into activated M1 or M2 phenotypes. M1-polarized TAMs are characterized by the expression of pro-inflammatory and anti-tumorigenic cytokines and chemokines, whereas M2-macrophages suppress antitumor immunity, contributing to PDAC progression [119,120]. Tumor cells can utilize sEVs to induce the differentiation of M1-TAMs towards a M2-immunosuppressive phenotype [121]. These M2-TAMs not only orchestrate immunosuppression, but also promote radiation- and chemoresistance, angiogenesis, migration, invasion as well as metastasis [122]. M2-polarization was reported upon uptake of PDAC-derived sEVs loaded with intercellular adhesion molecule-1 (ICAM-1) and arachidonic acid (AA), triggering the secretion of pro-angiogenic and pro-metastatic factors. To this end, ICAM-1 on sEVs interacted with CD11c on macrophages, which facilitated the secretion of pro-tumorigenic molecules and uptake of the respective sEVs was further enhanced by AA [123]. Patient-tumor-sEVs enriched in Ezrin (EZR) also directed M2-polarization in vivo, enhancing liver metastasis [124]. 

Vice versa, sEVs from M2-macrophages interacted with PDAC tumor cells as well as the extended TME, e.g., M2-derived sEVs with miR-501-3p inhibited transforming growth factor beta receptor 3 (TGFBR3), enabling TGF-β signaling, tumor growth, and metastasis of xenografted PDAC in nude mice. Interestingly, miR-501-3p is also highly expressed in PDAC patient tissue [125]. Moreover, sEVs derived from M2-macrophages containing miR-155-5p and miR-221-5p further promoted angiogenesis in vitro by targeting the E2F transcription factor 2 (E2F2). Uptake of the respective sEVs in mice additionally enhanced vascular density and growth of subcutaneous tumors [126]. Transfer of M2-macrophage-sEVs with miR-365 reduced sensitivity of PDAC cells to gemcitabine in vitro and in vivo, enhancing migration and invasion of PDAC cells by targeting B-cell translocation gene 2 (BTG2) and activating FAK/AKT signaling [127]. Thus, sEV-based crosstalk between PDAC tumor cells and TAMs has a major function in shaping an immunosuppressive, tumor supporting TME.

#### 5.4.2. Immunosuppression by Myeloid-Derived Suppressor Cells

MDSCs are important innate regulators of the immune response. They are a heterogenous group of immature myeloid cells with potent immunosuppressive activity [128,129]. In PDAC patients, MDSC frequency in the peripheral blood is associated with metastatic disease and poor clinical outcome [130]. According to their origin from either monocytic or granulocytic myeloid cell lineages, MDSCs are classified in two main subgroups: monocytic (M-MDSCs) or granulocytic/polymorphonuclear MDSCs (G/PMN-MDSCs) [129,131]. Upon persistent exposure to inflammatory signals and myeloid growth factors, MDSCs are activated and regulate a variety of immunological and non-immunological pro-tumorigenic functions, including immune evasion, angiogenesis, EMT, and PMN-formation [129,132]. There is even evidence that hypoxic conditions can stimulate the differentiation of MDSCs into M2-TAMs and that MDSCs in general may enhance their pro-tumorigenic activity [131,133,134]. Interestingly, MDSCs can be activated by PDAC-sEVs. PDAC cells lacking expression of the tumor suppressor SMAD4 were shown to release sEVs containing miR-1260a and miR-494-3p, which changed the balance between DCs and MDSCs towards a higher number of M- and G-MDSCs, thereby promoting proliferation, glycolysis, and immunosuppression. The expression of SMAD4 is lost in around 55% of PDACs and associated with a poor prognosis [135]. Thus, loss of the SMAD4 tumor suppressor in PDAC is associated with altering sEV secretion and cargo content to generate an immunosuppressive TME.

#### 5.4.3. Adaptive Immune Suppression-Targeting T-Cell Activation by DCs and T_regs_

In addition to the innate immune response, PDAC can also bypass the adaptive immunosurveillance utilizing sEVs. In the PDAC TME, DCs are scarce and more frequently detected at the edge of tumors. In patients, more circulating DCs were associated with improved survival [136]. DCs are a diverse population of antigen-presenting cells, which are key modulators of the adaptive immune response that promote antigen-specific immunity and tolerance [137]. DCs facilitate activation of CD8+ T_effector_ cells by presenting antigens and releasing immunomodulatory cytokines, such as interleukin-12 (IL-12) and type I interferons to drive antitumor immunity. Additional conditioning of the TME with chemokines, such as CXCL9 and CXCL10 further promotes T-cell attraction and recruitment. DCs also support CD4+ T-cell differentiation towards a T_helper_ type 1(T_h_1)-phenotype with antitumor functions [138]. Thus, reprogramming of DCs by tumor-sEVs is a key step in perturbing adaptive, but also innate anti-tumor responses, impairing T-cell efficiency, the expression of Toll-like receptors (TLRs) or interleukins (ILs) [138,139,140]. For example, uptake of miR-203 in PDAC-derived sEVs by DCs was reported to inhibit the expression of TLR4, tumor necrosis factor-α (TNF-α), and IL-12 and mediate DC dysfunction [140]. Moreover, transfer of PDAC-sEVs to DCs with mir-212-3p, inhibited the expression of the transcription-factor-regulatory-factor-x-associated protein (RFXAP) and mediated downregulation of MHC II receptors as well as failure of CD4+ T-cell activation [141].

In vivo studies further indicated that PDAC-derived sEVs inhibit IL-2-mediated signaling to lymphocytes upon uptake by DCs and macrophages, promoting lymphocyte apoptosis [142]. In line, direct uptake of PDAC-sEVs by leukocytes caused inhibition of proliferation and impaired anti-apoptotic signaling, as well as IL-12-induced T_h_-cell proliferation. Moreover, the respective PDAC-sEVs interfered with chemotaxis of leukocytes towards the tumor [142]. The TME of PDACs is also characterized by a high number of inhibitory T_regs_. T_regs_ are classified as a subset of CD4+ T-lymphocytes, which express the transcriptional regulator Forkhead-box-protein P3 (FOXP3). They are crucial modulators of the immune system, which help to maintain tolerance against self-antigens, and suppress T_effector_ cell activation as well as clonal expansion [143,144]. T_regs_ are already detected in, or near early PanINs. Their numbers expand with PDAC progression and elevated levels were associated with bad prognosis for patients [7,145]. A recent study showed that the increased number of T_regs_ is partly caused by PDAC-sEVs that foster T_reg_ expansion by enhanced expression of FOXP3 [146].

In summary, PDAC-sEVs are vitally implicated in shaping the PDAC TME and evading anti-tumor immune surveillance by the innate and adaptive immune system. However, sEVs can also enter the circulation to mediate effects over longer distances, such as faciliating organotropic metastasis. Circulating sEVs can even be utilized as a platform for biomarkers associated with diagnosis and prognosis of PDAC.

## 6. sEVs in PDAC Metastasis

Ample evidence has demonstrated that PDAC-derived sEVs not only act as extracellular signaling hubs for TME remodeling, but also help to shape and establish PMNs in distinct organs.

### 6.1. PDAC-Derived sEVs and Formation of Distant PMNs

PDACs are characterized by a high propensity to metastasize, as the majority of PDAC patients present with metastases at the time of diagnosis [2,82]. The main sites for PDAC metastasis are the liver and lungs, but also the peritoneal cavity [82]. The formation of tumor metastases in distinct organs is dependent on the establishment of suitable PMNs. PMNs facilitate cancer dissemination by supporting survival and spread of cancer initiating cells (CIC) [147]. Recently, sEVs which express specific integrin combinations, such as integrin avβ5, were reported to drive organ-specific metastasis, i.e., in the liver by facilitating the respective PMN formation with the help of resident cell populations. Integrins are important signaling mediators during metastasis, which mediate cell-ECM adhesion, mechano-signaling, and cell migration by acting as transmembrane receptors for various physiological extracellular ligands. The integrin expression pattern on the cells surface is therefore a key factor in determining the behavior of cells in response to microenvironmental cues. Once dysregulated, altered integrin expression has been linked to various steps during cancer progression, including priming of metastatic niches, extravasation, homing of CICs to distant sites, as well as metastatic colonization [148,149]. Interestingly, integrins are also vital sEV cargos and can be recycled from the cell surface via endocytosis into MVBs and eventually sEVs [150,151]. In many instances recycling and packaging of integrins into MVBs is dependent on their interaction with Tspans that are a major sEV cargo class [24,47,152]. During PDAC progression, sEVs with specific integrin expression patterns were shown to mediate PMN formation in liver and lungs [153,154]. Costa-Silva et al. reported, that PDAC-sEVs crucially contribute to liver metastasis by transferring migration inhibitory factor (MIF) to Kupffer cells (KCs) in the liver. This resulted in increased TGF-β expression by KCs, which in turn activated hepatic stellate cells (HSCs) to secret fibronectin and induce the expression of proinflammatory mediators to facilitate formation of a suitable liver niche. This was corroborated in PDAC patients with liver metastases, which presented with elevated levels of MIF-positive plasma sEVs as compared to healthy control subjects, or patients with 5-year progression-free PDAC [153]. As described above, PDAC-sEVs positive for integrin αvβ5 were shown to facilitate the establishment of pre-metastatic liver niches, whereas integrins α6β4- or α6β1 directed niche formation and metastases in the lung [154]. In line, our group has recently demonstrated that Protein kinase D1 (PRKD1) expression was significantly downregulated in many PDACs, compared with non-tumor tissue. Loss or inhibition of PRKD1 strongly enhanced sEV release from different PDAC cells and changed the expression of integrins in cells as well as secreted sEVs to high levels of integrins α6β4, while impairing expression of integrin β5. Thus, injection of PRKD1^KO^-sEVs effectively enhanced lung metastasis of Panc-1 cells in xenografted mice. We have also demonstrated that the enhanced expression of integrins α6β4 in PRKD1^KO^-sEVs was facilitated by transcriptional upregulation in cells, as well as increased endosomal recycling and packaging of integrin α6β4 from the cell surface into sEVs in a Tspan CD82-dependent manner. Moreover, autochthonous Prkd1 knockout mice in a Kras^G12D^ background showed predominant lung and no visible liver metastasis. This may be attributed to the abrogated formation of integrin αvβ5 dimers due to low levels of integrin β5 in PRKD1^KO^-cells and -sEVs. The PMN in the lung was ultimately established upon uptake and reprogramming of PRKD1^KO^-sEVs by lung fibroblasts, which induced expression of proinflammatory regulators S100A6, A13, and A16. To this end, transfer of respective s100a mRNAs by sEVs was also suggested [47]. In summary, these data indicate that PDAC do not only utilize their sEVs to communicate in the local TME, but also over long distances via the blood flow to establish PMNs and facilitate subsequent PDAC dissemination. 

### 6.2. sEVs in PDAC Tumor Proliferation, EMT, Invasion, and Metastasis

Once PMNs at distant organs have been established, PDAC cells need to acquire a motile, invasive phenotype, e.g., undergoing EMT to subsequently enter the circulation and metastasize. During EMT, tumor cells loose epithelial features, such as E-cadherin expression and acquire a mesenchymal phenotype by expressing vimentin, fibronectin and N-cadherin as well as MMPs [155,156]. EMT is controlled by zinc-finger transcription factors, such as SNAIL, SLUG, and TWIST downstream of growth factor signaling, e.g., EGF, TGFβ, or Wnt/β-catenin pathways [157,158,159,160]. PDAC-sEVs affect these processes in an auto- and paracrine manner. PDAC-derived sEVs containing Tenascin-c (TNC) were described to drive PDAC migration, invasion, and EMT by mediating Wnt/β-catenin signaling. TNC-containing PDAC-sEVs also increased PDAC proliferation by activating NF-κB [161]. PDAC metastasis is further regulated by signaling through miRNAs. The RNA-binding protein LIN28B was shown to promote PDAC growth and metastasis by inhibiting the biogenesis of let-7 family miRs [162]. In PDAC tumor-bearing xenografted mice, injection of LIN28B-positive sEVs activated LIN28B/let-7/high-mobility group AT-hook 2 (HMGA2)/platelet derived growth factor subunit B (PDGFB) signaling to facilitate PDAC liver metastasis [162].

Moreover, blood vessels are attracted to tumors, by inducing neoangiogenesis to secure oxygen/nutrient supply and foster metastatic dissemination. As detailed in Section 5.3, angiogenesis of blood and lymphatic vessels is facilitated by sEVs, yet this process is not only limited to the TME but also supports tumor growth at metastatic sites [163]. Once metastases are established at distant organs, prognosis for patients is exceedingly bad. This is even further aggravated when PDAC tumors have acquired additional resistance against radio- and chemotherapies [5,164].

## 7. sEVs in Chemoresistance

Resistance towards chemotherapy is a major limiting factor for curative treatment of PDAC patients. Chemoresistance is multifactorial, and depends on parameters, such as tumor burden, tumor heterogeneity, physical barriers due to fibrosis, the immune system as well as undruggable cancer drivers [165]. Gemcitabine (GEM) chemotherapy is one of the agents used as standard of care for PDAC treatment and resistance towards GEM is a severe problem, reducing the efficacy of the response in advanced or metastatic disease [166]. Tumor cells have adopted different resistance mechanisms to evade chemotherapy, including sEVs. For example, paracrine transfer of miRNAs to surrounding PDAC cells by PDAC-derived sEVs facilitates chemoresistance. GEM-resistant PDAC cells were shown to transduce drug resistance to non-resistant cells by sEV-based transfer of miR-210, which activated the mTOR pathway in vitro and in vivo, and treatment of non-resistant cells with the respective sEVs also stimulated their proliferative and anti-apoptotic capacities [167]. Moreover, miR-155-loaded PDAC-sEVs from drug resistant cells transduced drug resistance by downregulation of deoxycytidine kinase (DCK), a GEM-metabolizing enzyme. PDAC patients with high levels of miR-155 in PDAC tissue were further reported to have a poor prognosis [168]. In addition, transfer of transcripts for ROS-detoxifying superoxide dismutase 2 (SOD2) and catalase (CAT) by GEM-resistant-PDAC-sEVs caused increased expression of the respective mRNAs, impairing GEM-mediated ROS production [169]. Thus, sEVs are potent regulators of chemoresistance in PDAC. 

Table 1 represents a summary of sEV cargos and their respective functions in PDAC.

## 8. sEVs as Biomarkers for Prognosis and Prediction

Besides a prominent role of circulating PDAC-sEVs in facilitating the formation of PMNs and systemic chemoresistance, the respective sEVs have also been proposed as effective biomarker platforms, accessible by blood liquid biopsy. It is thus tempting to utilize sEVs and their cargos for early detection of PDAC and their differentiation from more benign pancreatic diseases, such as pancreatitis. In this context, sEV-resident glypican-1 (GPC-1) is one of the most studied PDAC markers to date. It was originally discovered by Melo et al. using animal and human cell lines and presented with a sensitivity/specificity of 100%, upon detection by transmission electron microscopy on sEVs. ELISA detection reduced the sensitivity and specificity to 82.14% and 75%, respectively [191]. Further validation of these findings using alternative sEV purification techniques after sampling sEVs from peripheral or portal vein blood has demonstrated a sensitivity of 64%, whereas the specificity was 90%. This was still more sensitive than fine needle biopsy and the current gold-standard maker carbohydrate antigen 19-9 (CA19-9). In their hands, the best diagnostic accuracy was obtained when all three methods were combined or by using GPC-1-sEVs together with serum CA19-9 [192]. However, it has to be noted that a validation attempt for GPC-1, as part of another study using ELISAs to detect sEVs, identified no significant difference for PDAC patient samples in respect to benign pancreatic conditions. Thus, further validation and standardization of sEV purification as well as detection methods are required to achieve reproducible results for broad clinical diagnostic use [193]. A challenge to identify viable biomarkers is the reliable differentiation of early-stage cancer from benign pancreatic processes. To this end, EphA2 has been tested as possible biomarker in sEVs, which achieved a sensitivity and specificity of 91% and 85%, respectively, for identifying stage I and II PDAC compared with healthy controls. Moreover, it was also possible to utilize EphA2 to differentiate stage I and II PDAC from pancreatitis with a sensitivity/specificity of 86%/85%, respectively [110]. Others used multiple biomarkers to increase specificity for the detection of PDAC. To this end, a PDAC-sEV marker panel with EGFR, EpCAM, HER2, mucin-1 (MUC1), GPC-1, and Wnt family member 2 (WNT2) was described that reached a sensitivity of 95%, specificity of 81%, and accuracy of 88% in a prospective cohort of 43 subjects [194]. In addition to proteins also sEV-resident miRNAs were investigated as a potential diagnostic tool for the early detection of PDAC. Serum-derived sEVs from PDAC patients were reported to contain elevated levels of miR-192-5p, miR-19a-3p, and miR-19b-3p, when compared to healthy controls [195,196]. Additional studies described significantly more miR-10b, miR-17-5p and miR-21 in PDAC patient samples [197,198,199,200]. Interestingly, miR-21 was also increased in different solid tumors, suggesting a common mechanism involved in carcinogenesis, yet this also limits its use as a PDAC-specific biomarker [201]. 

sEVs are not only proposed for diagnostic use, but also to identify markers with prognostic value. It has been shown that increased levels of ANXA6-positive sEVs correlate with poor prognosis [187]. Furthermore, programmed death-ligand 1 (PD-L1) on sEVs was used as a prognostic marker, associating high levels with significant shorter average post-resection survival times [202]. The sEV cargo EpCAM was also investigated as a prognostic marker. Analysis of sEVs from patients with metastatic or non-resectable locally advanced PDAC indicated higher levels of EpCAM correlated with shorter progression-free and overall survival [203]. 

A major prognostic factor for PDAC is metastasis. PDAC-derived sEVs were described to induce profibrogenic activities to facilitate the formation of PMNs. This has been demonstrated by Costa-Silva et al., whereby sEVs with MIF helped to establish PMNs in the liver, as described in Section 6.1 [153]. Moreover, the amount of GPC-1 in sEVs was positively correlated with distant metastasis [191]. Thus, sEV-based biomarker analysis has the potential to develop into a potent tool for clinical use. Advantages of sEVs include protection of protein cargos from proteolytic cleavage as well as preventing the degradation of nucleic acids [30]. Moreover strategies have been developed to enrich tumor-specific sEVs by immune purification to increase specificity and sensitivity of sEV analyses [204]. Castillo et al. have identified a set of sEV surface markers: Claudin 4 (CLDN4), EPCAM, CD151, Galectin 3 Binding Protein (LGALS3BP), and Histone H2B type 2-E and F (HIST2H2BE, HIST2H2BF) to enrich PDAC-specific sEVs after liquid biopsy, thus enabling a more sensitive detection of mutated KRAS [205]. We therefore suggest that similar strategies may be employed to improve the analysis of other diagnostic or prognostic cargos. A comprehensive summary of diagnostic and prognostic sEV biomarkers for PDAC is presented in Table 2. 

## 9. Therapeutic sEVs

The use of sEVs as therapeutic vehicles is still in its early development. A promising study by Kamerkar et al. in 2017 modified sEVs from fibroblast-like mesenchymal cells with siRNAs or shRNAs against mutated and wildtype KRASG12D (iExosomes). Subsequent sEV-treatment of mice with PDAC tumors in a Kras^G12D^ background for 30 days demonstrated a significant reduction in tumor size in respect to the untreated control mice. A comparison to liposomes loaded with the same cargo further indicated superior size reduction in the iExosome treatment group. Interestingly, these effects were even evident after 200 days of treatment and survival of mice was significantly increased. This concept is currently also evaluated in a Phase I clinical trial in PDAC patients with a KRAS^G12D^ mutation (NCT03608631) [217]. In another study, paclitaxel-containing sEVs from MSCs were shown to reduce PDAC cell proliferation [218]. Recently, sEVs derived from bone marrow mesenchymal stem cells (BM-MSCs) were loaded with a combination of siRNA against galectin-9 and engineered to carry oxaliplatin (OXA) prodrug on their surface (iEXO-OXA). Galectin-9 was used to block Galectin9/dectin-1 signaling to overt immunosuppression by M2 macrophages, whereas the chemotherapeutic agent OXA-prodrug was introduced to trigger immunogenic PDAC cell death (ICD). In vivo-treatment of established Panc-02 tumors using these iEXO-OXA nanoparticles thus effectively stimulated innate and adaptive anti-tumor immune responses, enhanced ICD and infiltration by cytotoxic T-lymphocytes as well as promoted DC maturation [219]. 

However, there are still many improvements required concerning targeting of engineered sEVs to specific cell populations by utilizing either natural tropism of sEVs or in promoting the development of sEV modification strategies, yet initial research is promising and may help to offer novel treatment avenues for PDAC.

## 10. Conclusions and Perspectives

**Figure 1:**
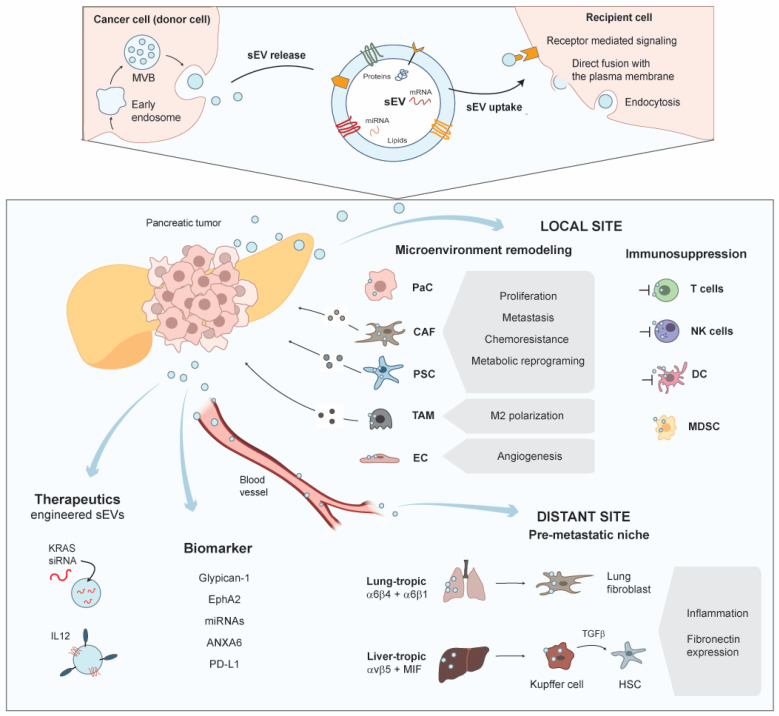
sEVs in short- and long-distance intercellular communication during PDAC initiation, progression, and metastasis.

In this review, we have discussed the roles of sEVs (exosomes) in PDAC initiation, tumor growth, progression, angiogenesis, immune evasion, and metastasis. Extensive research in the last years has indicated that PDACs are characterized by an extensive crosstalk via secretion of sEVs with the cellular components of their TME. There is ample evidence that sEV-based interactions between PDAC cells and CAFs or PSCs, TAMs, T-cells as well as other immune cells (Section 5) regulate tumor growth, chemoresistance, immune evasion, and invasiveness (Section 5, Section 6 and Section 7, Figure 1). Interestingly, these interactions are not only limited to short-range communication in the TME, but also complemented by a vital role of sEVs in the establishment of distant PMNs by the distribution of sEVs through the blood circulation (Section 6.1). Thus, sEVs function as vital signaling hubs during PDAC progression and metastasis. Different sEV cargo classes facilitate the reprogramming of target cells, which include lipids, proteins, miRNAs, mRNAs, but also long non-coding or circular RNAs. The respective cargos and their roles in PDAC evolution are summarized in Table 1. Additionally, there is increasing evidence that circulating sEVs may be utilized as effective biomarker platforms for diagnosis or prognosis. To this end, different combinations of markers and cargo classes have been evaluated to classify disease states or treatment responses (Table 2). However, there is still extensive research needed to establish minimal classifiers that are sensitive and specific enough for adoption in routine clinical use. One major problem of liquid biopsies is the diverse origin of sEVs present in the circulation. Although tumor cells are known to secrete a large amount of sEVs [86], other cells significantly contribute sEVs and cargos to the sampled biopsies. Thus, it would be advantageous to purify or enrich tumor-specific sEVs from the circulation. First steps have been taken in this direction utilizing immuno-enrichment of sEVs [204,205] (Section 8), however sensitivity and specificity still need to be optimized for routine clinical use. There are also studies that have used sEVs as therapeutic vehicles [217,218] (Section 9). This is an exciting and promising use-case. To exploit a therapeutic function for sEVs, the nanovesicles need to be extensively modified during biogenesis or post-release. Moreover, specific targeting will be required [220,221] and the cargo composition of sEVs will need to be specifically adapted. There are already studies on the way to optimize the methodology for these modifications, which even include the generation of artificial engineered sEVs [222]. Yet considering the challenges, extensive research will be needed before such applications will be ready for routine clinical use. Nevertheless, sEV research over the last years has greatly contributed to a better understanding of the complex mechanisms that drive PDAC initiation, progression, and metastatic dissemination and will hopefully soon translate into practical therapeutic options.

## Figures and Tables

**Table 1:** sEV cargos and their respective functions in PDAC.

Biological Process	Donor Cell	Recipient Cell	sEV Cargo	Function in PDAC	Reference
Precancerous diseases (PD)	PSCs	PSCs	miR-21-5p	miR-21-5p regulates CCN2 expression, facilitating proliferation and collagen deposition	[76]
	MSCs	PACs	Klotho	Attenuates caerulein- induced activation of NF-κB, stimulating growth and apoptosis resistance	[170]
	hPDAC cells	DCs	miR-212-3p	Inhibition of RFXAP, causing MHC II downregulation and CD4+ T-cell activation (also relevant in IS and MET)	[141]
	hPDAC cells	DCs	miR-203	Inhibition of DC function by suppressing TLR4, TNF-α, and IL-12 expression (also relevant in IS)	[140]
	hPDAC cells	MDSCs	miR-1260a	Reprogramming of g/mMDSCs, bolstering proliferation and glycolysis, thus establishing a immunosuppressive TME (also relevant in IS)	[135]
	rPDAC cells	rPDAC cells	CD151	Induction of EMT and migration	[171]
	rPDACCIC	rPDAC cells	Cld7	Reprogramming of non-metastatic cells to increase their invasiveness (also relevant in AG and MET)	[172]
Immunosuppression (IS)	hPDAC cells	Macrophages	ICAM-1/AA	ICAM-1 interacts with surface-exposed CD11c on macrophages promoting M2 polarization, triggering angiogenesis and metastasis. AA facilitates sEV-uptake by macrophages.	[123]
	hPDAC cells	Macrophages	miR-301a-3p	(also relevant in MET)	[90]
	hPDAC cells	Macrophages	EZR	M2 polarization of macrophages, promoting liver metastasis	[124]
	hPDAC cells	DCs	miR-212-3p	(also relevant in PD and MET)	[141]
	hPDAC cells	DCs	miR-203	(also relevant in PD)	[140]
	hPDAC cells	DCs	miR-1260a	(also relevant in PD)	[135]
	Patient plasma sEVs	BCs	TAA	Trapping of anti-TAA-antibodies and complement-mediated cytotoxicity, preventing B-lymphocytes from properly engaging tumors	[173]
	hPDAC cells	T lymphocytes	FOXP3	Enhanced sEV-induced FOXP3 expression and T_reg_ expansion mediated by the ATM-AMPK-SIRT1/2/6-FOXO1A/FOXO3A axis, resulting in impaired anti-tumor immunity of T lymphocytes against PDAC cells	[146]
Angiogenesis (AG)	rPDAC cells	EC	Tspan8/106/49d	VEGF-independent regulation of angiogenesis-related genes, triggering EC proliferation, maturation of EC progenitors, migration and sprouting	[107]
	hPDAC cells	HMVEC	miR-27a	Suppression of BTG2, inducing proliferation, migration and angiogenesis	[174]
	hPDAC cells	EC	Circ-IARS	Increase of endothelial cell permeability and angiogenesis, promoting invasiveness. Downregulation of miR-122 and ZO-1 as well as upregulation of active RhoA-GTP and F-Actin, contributing to PDAC invasion (also relevant in MET)	[175]
	M2 macrophages	EC	miR-155-5p miR-221-5p	Targeting of E2F2 enhances vascular density and tumor growth	[126]
	hPDAC cells	EC	VEGF-C	Downregulation of DUSP-2 facilitates release of VEGF-C-containing sEVs, resulting in lymphovascular invasion	[108]
	rPaCIC	rPDAC cells	Cld7	(also relevant in PD and MET)	[172]
Proliferation	hPDAC	PSCs	miR-1246 miR-1290	Upregulation of α-SMA, production of PIP and activation of ERK, Akt signaling, inducing proliferation and migration	[99]
	hPDAC cells	PHFF	mRNA-hTERT	Transformation of non-malignant pancreatic fibroblasts, delaying aging and stimulating proliferation	[176]
	hPDACSCs	hPDAC cells	miR-210	Activation of mTOR pathway, stimulating proliferation and apoptosis resistance	[167]
	PSCs	PSCs	miR-21-5p	(also relevant in PD)	[76]
	PSCs	hPDAC cells	miR-5703	Targeting of CMTM4, promoting proliferation due the activation of PI3K/Akt pathway by PAK4	[101]
	CAFs	hPDAC cells	de novo metabolites	Reprogramming the energy metabolism of PDAC cells, enhancing the Warburg effect, promoting growth and survival	[91]
Metastasis (MET)	hPDAC cells	hPDAC cells	CD44v6	Activation of Wnt/β-catenin signaling, increasing expression of PAI-1, MMPs and TIMP-1, enhancing cell migration and metastasis. Promotes motility and invasion by interacting with integrins and proteases	[177]
	rPDAC cells	rPDAC cells	CD151/Tspan8	Increase in expression of proinflammatory regulators and EMT-associated transcripts as well as promotion of ECM remodeling, fostering angiogenesis and metastasis	[171]
	rPaCIC	rPDAC cells	Cld7	(also relevant in PD and AG)	[172]
	hPDAC cells	hPDAC cells/PSCs	Lin28B	Inhibition of let-7 family miR-biogenesis, promoting growth and liver metastasis. Promotion of PSC recruitment by upregulating PDGFB resulting in the activation of the Lin28B/let7/HMGA2/PDGFB signaling pathway	[178]
	m/hPDAC cells	KCs HSCs	MIF	Stimulation of TGF-β by KCs, triggering fibronectin production of HSCs, fostering pre-metastatic niche formation in the liver	[153]
	hPDAC cells	hPDAC cells	Plectin	Promotion of proliferation, migration, and invasion	[179]
	hPDAC cells	hPDAC cells	ZIP4	Promotion of proliferation, migration, and invasion	[180]
	CM/serum	hPDAC cells	miR-222	Impaired expression, phosphorylation and nuclear exit of p27 via PPP2R2A/Akt, promoting proliferation and invasiveness	[181]
	hPDAC cells	Macrophages	miR-301a-3p	M2 polarization of macrophages and HIF1α/2α-promoted activation of PI3K-signaling, fostering survival, proliferation, and metastasis (also relevant in IS)	[90]
	mPDAC cells	mPDAC cells	miR-339-5p	Downregulation of ZNF689, inhibiting migration and invasion	[182]
	Macrophages	hPDAC cells	miR-501-3p	Inhibition of TGFBR3 and activation of TGF-β signaling, inducing growth, and metastasis	[125]
	hPDAC cells	EC/HUVEC	Circ-IARS	(also relevant in AG)	[175]
	hPDAC serum	hPDAC cells	Circ-PDE8A	Counteracting of miR-338 activates MACC/MET/ERK/Akt signaling, inducing invasive growth	[183]
	hPDAC cells	Lung fibroblasts	Integrin α6β4 Integrin α6β1	Lungtropic metastasis Packaging of α6β4 into sEVs in a CD82-dependent manner in cells with loss of PRKD1	[47,154]
	hPDAC cells	Macrophages	EZR	M2 polarization of macrophages, triggering metastasis	[124]
	hPDAC cells	KC	Integrin αvβ5	Livertropic metastasis	[153]
	hPDAC cells	hPDAC cells	miR-23b-3p	Promotion of proliferation, migration and invasion	[184]
	hPDAC cells	DCs	miR-212-3p	(also relevant in PD and IS)	[141]
	hPDAC cells	hPDAC cells	VEGF-C	(also relevant in AG)	[108]
	hPDAC cells	hPDAC cells	miR-125b-5p	Inhibition of STARD13, enhancing EMT as well as migration and invasion	[185]
	hPDAC cells	hPDAC cells	lnc-Sox2ot	Competitive binding to miR-200 family upregulates Sox2 expression, inducing EMT and stem cell-like properties of PDAC cells, thus contributing to invasion and metastasis	[186]
	CAFs	hPDAC cells	ANXA6/LRP1/TSP1	Increased PDAC aggressiveness and metastasis	[187]
Chemoresistance (CR)	CAFs	hPDAC cells	Snail miR-146a	Promotion of survival, proliferation and drug resistance	[188]
	CAFs	hPDAC cells	miR-106b	Downregulation of TP53INP1, promoting proliferation and drug resistance	[189]
	hPDAC cells	hPDAC cells	miR-155	Downregulation of DCK or upregulation of ROS-detoxifying genes SOD2 and CAT, promoting drug resistance	[169]
	M2 macrophages	hPDAC cells	miR-365	Upregulation of triphospho-nucleotide pool in PDAC cells, induction of cytidine deaminase activation or targeting of BTG2 to stimulate FAK/AKT pathway, triggering drug resistance	[127]
	hPDAC cells	hPDAC cells	EphA2	Promotion of drug resistance	[190]

**Table 2:** sEV biomarkers for diagnosis and prognosis of PDAC.

Source	sEV Cargo	Diagnostic/Prognostic Function	Reference
Plasma Serum	miR-16 miR-196a CA19-9	(Early) diagnosis	[206]
Serum	miR-20a miR-21 miR-24 miR-25 miR-99a miR-185 miR-191	Diagnosis and prognosis	[207]
Serum	miR-1290	(Early) diagnosis	[208]
Serum	miR-17-5p	Diagnosis	[198]
Serum	miR-21	Diagnosis	[198]
Portal vein blood		Recurrence and prognosis	[199]
Pancreatic juice		Diagnosis	[200]
Plasma	miR-10b	Diagnosis	[209]
Plasma	High miR-10b miR-21 miR-30c miR-181a Low miR-let7a	Diagnosis	[197]
Plasma	miR-196a	Diagnosis	[210]
Plasma	miR-122-5p miR-125b-5p miR-192-5p miR-193b-3p miR-221-3p miR-27b-3p	Diagnosis and prognosis	[211]
Portal vein blood	miR-451a	Recurrence and prognosis	[199]
Pancreatic juice	miR-155	Diagnosis	[200]
Serum	mir-1226	Diagnosis and prognosis	[212]
Serum	miR-1246 miR-4644 miR-3976 miR-4306 CD44v6 Tspan8 EpCAM MET CD104	Diagnosis	[213]
Plasma	MIF	Prognosis	[153]
Serum	GPC1	Diagnosis and prognosis	[191]
Plasma	EGFR EpCAM MUC1 GPC1 WNT2	Diagnosis	[194]
Plasma	EphA2	Diagnosis	[110]
Plasma	EGFR CA19-9	Proposed to have diagnostic potential	[214]
Serum	CKAP4	Diagnosis and monitoring	[215]
Serum	c-MET	Prognosis	[202]
Serum	PD-L1	Prognosis	[202]
Plasma	CLDN4 EpCAM CD151 LGALS3BP HIST2H2BE HIST2H2BF	Surface marker for enrichment of PDAC-sEVs	[205]
Plasma	EpCAM	Prognosis	[203]
Circulating sEVs	CD44v6	Prognosis	[216]
Circulating sEVs	C1QBP	Prognosis	[216]
Serum	ANXA6	Potential biomarker	[187]
Plasma	lnc-Sox2ot	Prognosis	[186]
